# Effect of screw threading dislocations and inverse domain boundaries in GaN on the shape of reciprocal-space maps^1^

**DOI:** 10.1107/S1600576717003612

**Published:** 2017-03-22

**Authors:** Mykhailo Barchuk, Mykhaylo Motylenko, Gleb Lukin, Olf Pätzold, David Rafaja

**Affiliations:** a Institute of Materials Science, TU Bergakademie Freiberg, Gustav-Zeuner-Strasse 5, Freiberg 09599, Germany; b Institute of Nonferrous Metallurgy and Purest Materials, TU Bergakademie Freiberg, Leipziger Strasse 34, Freiberg 09599, Germany

**Keywords:** gallium nitride, X-ray diffraction, transmission electron microscopy, threading dislocations, inversion domain boundaries

## Abstract

The microstructure of polar GaN layers, grown by upgraded high-temperature vapour phase epitaxy on [001]-oriented sapphire substrates, was studied by means of high-resolution X-ray diffraction and transmission electron microscopy. Systematic differences between reciprocal-space maps measured by X-ray diffraction and those which were simulated for different densities of threading dislocations revealed that threading dislocations are not the only microstructure defect in these GaN layers. Conventional dark-field transmission electron microscopy and convergent-beam electron diffraction detected vertical inversion domains as an additional microstructure feature. On a series of polar GaN layers with different proportions of threading dislocations and inversion domain boundaries, this contribution illustrates the capability and limitations of coplanar reciprocal-space mapping by X-ray diffraction to distinguish between these microstructure features.

## Abstract 2

Polar GaN layers containing domains with inverse polarities are studied by means of high-resolution X-ray diffraction and transmission electron microscopy. It is shown how the presence of inversion domain boundaries can be recognized directly from reciprocal-space maps measured by X-ray diffraction.

## 1. Introduction   

The physical properties of gallium nitride (GaN) make this material one of the most promising direct wide-bandgap semiconductors for optoelectronics and high-power and high-frequency devices. GaN single crystals are typically grown by hetero-epitaxy on foreign substrates, *e.g.* Al_2_O_3_, SiC, Si *etc.* (Nakamura & Fasol, 1997 ▸; Jain *et al.*, 2000 ▸). As a rule, hetero-epitaxial deposition processes produce a large number of microstructure defects that stem mainly from a mismatch between the lattice parameters of the layer and the respective substrate.

In polar [001]-oriented GaN, the dominant microstructure defects are threading dislocations (TDs). Their density, which varies between 10^5^ and 10^10^ cm^−2^ (Hino *et al.*, 2000 ▸; Motoki *et al.*, 2002 ▸; Xu *et al.*, 2002 ▸; Datta *et al.*, 2004 ▸; Hertkorn *et al.*, 2008 ▸; Booker *et al.*, 2010 ▸; Mynbaeva *et al.*, 2016 ▸), is one of the most important factors downgrading the electronic properties of GaN layers (Speck & Rosner, 1999 ▸). One of the common techniques used to detect TDs and quantify their density is X-ray diffraction (XRD). In [001]-oriented GaN layers, the density of screw TDs can be determined from the broadening of the 00*l* reflections in the azimuthal direction (Metzger *et al.*, 1998 ▸; Chierchia *et al.*, 2003 ▸; Srikant *et al.*, 1997 ▸; Safriuk *et al.*, 2013 ▸) or from the extent of diffuse scattering that arises around the 00*l* reciprocal-lattice points (Holý *et al.*, 2008 ▸; Barchuk *et al.*, 2010 ▸; Kaganer *et al.*, 2005 ▸, 2009 ▸). Diffuse scattering around asymmetric *hkl* reflections is also sensitive to the density of edge TDs (Barchuk *et al.*, 2010 ▸; Sun *et al.*, 2002 ▸; Kopp *et al.*, 2014 ▸).

Additionally, the XRD lines are broadened if the crystallite size is limited. Within the kinematical diffraction theory (Warren, 1990 ▸), the line broadening resulting from the limited crystallite size, *i.e.* from the small size of coherently diffracting domains, can be described by the Fourier transform of the crystallite shape. A finite lateral size of crystallites, *i.e.* a finite lateral correlation length, broadens the diffraction lines along the *x* and *y* directions of reciprocal space (*q*_||_). Within the conventional mosaicity model (Metzger *et al.*, 1998 ▸; Chierchia *et al.*, 2003 ▸), these two contributions to line broadening (due to dislocations and small crystallites) are distinguished by employing a modified Williamson–Hall plot (Williamson & Hall, 1953 ▸; Dunn & Koch, 1957 ▸).

In hetero-epitaxial micrometre-thick GaN layers grown on foreign substrates, the lateral correlation length is usually assumed to be relatively large, and thus the TDs are considered to be the sole source of diffraction line broadening (Barchuk *et al.*, 2010 ▸, 2014 ▸; Romanitan *et al.*, 2017 ▸). However, such layers can be composed of vertical domains with inverse polarities that are separated by inversion domain boundaries (IDBs). The domain sizes and IDB densities depend strongly on the specific substrate and epitaxial technique used for the GaN deposition (Potin *et al.*, 1997 ▸; Potin, Ruterana & Nouet, 1999 ▸; Ruterana, 2005 ▸; Romano *et al.*, 1996 ▸; Daudin *et al.*, 1997 ▸; Mogilatenko *et al.*, 2008 ▸).

Ruterana *et al.* (2000 ▸) have shown that small hexagonal terraces on Al_2_O_3_ substrates forming steps, which are parallel to the {110} planes, facilitate the growth of vertical IDBs in hetero-epitaxial GaN. These IDBs were found to cause a relative displacement of *r* = −*c*/8 (sometimes called IDB*) or *r* = 3*c*/8 (known as Holt-type IDBs), where *c* is the out-of-plane lattice parameter of GaN (Dimitrakopulos *et al.*, 2001 ▸; Koukoula *et al.*, 2014 ▸; Holt, 1969 ▸). The IDB* form is observed much more frequently by transmission electron microscopy (TEM) than the Holt-type IDBs (Ruterana, 2005 ▸; Dimitrakopulos *et al.*, 2001 ▸; Koukoula *et al.*, 2014 ▸; Potin, Nouet & Ruterana, 1999 ▸; Komninou *et al.*, 2001 ▸) because the latter require a higher formation energy. The Holt-type IDBs are electrically active, giving rise to electron–hole recombination (Dimitrakopulos *et al.*, 2001 ▸; Northrup *et al.*, 1996 ▸). The control of surface polarity is one of the most important issues in the development of nitride-based devices. The spontaneous occurrence of domains with Ga-face (+*c*) polarities in the N-face (−*c*) matrix is regarded as an undesired feature of GaN layers that complicates their use in optoelectronics (Sumiya & Fuke, 2004 ▸).

Domains with inverse polarities are typically studied using TEM (Potin *et al.*, 1997 ▸; Potin, Ruterana & Nouet, 1999 ▸; Ruterana, 2005 ▸; Romano *et al.*, 1996 ▸; Daudin *et al.*, 1997 ▸; Mogilatenko *et al.*, 2008 ▸; Dimitrakopulos *et al.*, 2001 ▸, 2005 ▸; Koukoula *et al.*, 2014 ▸). Kirste *et al.* (2011 ▸) performed temperature-dependent photoluminescence measurements and complemented them with Raman spectroscopy in order to investigate the structural and optical properties of lateral polarity junctions between Ga-face and N-face domains. Kemper *et al.* (2011 ▸) studied inversion domains in cubic GaN by applying a combination of atomic force microscopy, electron backscatter diffraction, and micro-Raman, photoluminescence and cathodoluminescence spectroscopies. Additionally, they used high-resolution X-ray diffraction (HRXRD) to estimate the density of the hexagonal inclusions that are considered defects in a cubic crystal, which changes the polarity locally. Heinke *et al.* (2001 ▸) detected IDBs in polar GaN by means of TEM and discussed the X-ray diffuse scattering from them in symmetric reflections. They also found good agreement between the lateral extensions of domains obtained from TEM and the defect correlation length estimated from the width of the diffuse scattering. Recently, Labat *et al.* (2015 ▸) determined the polarities of particular inversion domains, as well as local strains in the vicinity of IDBs, inside nanometre-sized GaN wires using coherent Bragg imaging. To the best of our knowledge, the effect of inversion domains in GaN on the shape of the reciprocal-space maps (RSMs) measured by XRD has not yet been reported.

In order to illustrate this effect, we deposited a series of polar GaN layers on [001]-oriented Al_2_O_3_ substrates that have different densities of IDBs, and measured the 004 and 114 RSMs of GaN. The density of the inversion domains was varied through the growth conditions in a reactor designed for high-temperature vapour phase epitaxy (Lukin *et al.*, 2014 ▸). The lateral width of the inversion domains was obtained from TEM experiments (dark-field TEM and convergent-beam electron diffraction).

## 2. Experimental   

A series of polar GaN samples were deposited on [001]-oriented sapphire substrates using a recently developed reactor designed for high-temperature vapour phase epitaxy (HTVPE) (Lukin *et al.*, 2014 ▸). The main advantage of this reactor is the variability of the deposition parameters over a broad parameter range. This characteristic of the HTVPE reactor was recently used to investigate the effect of ammonia flow on the formation of microstructure defects in GaN layers (Barchuk *et al.*, 2016 ▸). In this work, the GaN layers were grown at different reactor pressures.

Prior to HTVPE growth at the substrate temperature of 1373 K, a 50 nm thick nucleation layer was deposited on the sapphire substrate and annealed for 5 min at 1373 K in an argon/nitro­gen carrier gas containing additionally 0.4 s.l.m. (standard litres per minute) hydrogen. The recrystallized nucleation layers were overgrown in an argon/nitro­gen atmosphere. The actual GaN layers were deposited at reactor pressures of 150 mbar (1 bar = 100 000 Pa) (sample S1), 985 mbar (sample S2) and 200 mbar (sample S3), and at NH_3_ flows of 0.2 s.l.m. (samples S1 and S2) and 0.4 s.l.m. (sample S3). During the deposition of sample S3, 20% H_2_ was added into the carrier gas. Growth in the hydrogen-containing atmosphere is expected to reduce the density of TDs, to delay the coalescence of nucleation islands and consequently to facilitate the formation of inversion domains. The reactor pressure did control the growth rates, which were estimated at 19.6, 3.4 and 4.7 µm h^−1^ for samples S1, S2 and S3, respectively. The deposition times were adjusted to achieve almost the same thicknesses (around 4 µm) in all samples.

The X-ray diffraction measurements were carried out on a triple-axis Seifert/FPM diffractometer equipped with an Eulerian cradle, a sealed X-ray tube with a copper anode, and two dislocation-free (111)-oriented Si crystals. One Si crystal served as monochromator in the primary beam and the other as analyser of the diffracted beam. The cross section of the primary X-ray beam was adjusted to 0.09 × 2 mm. The instrumental broadening of the diffractometer was estimated to be 10′′ (Barchuk *et al.*, 2014 ▸). For each sample, the RSMs around reciprocal-lattice points (RLPs) 004 and 114 were collected as a set of radial (ω–2θ) scans, which were performed at different angles between the diffraction vector and the sample surface perpendicular direction (ψ). Additionally, azimuthal scans through reflections 002, 004 and 006 were recorded. All measurements were carried out in coplanar diffraction geometry.

The TEM investigations were done in a JEOL JEM-2200FS transmission electron microscope equipped with a field-emission electron gun, a Cs corrector located in the primary beam and a highly sensitive 2 k × 2 k CCD camera. The [100]-oriented cross-sectional TEM specimens were prepared using a precision ion polishing system (PIPS). The bright-field (BF) and dark-field (DF) TEM images and selected-area (SAED) and convergent-beam (CBED) electron diffraction patterns were processed using the *Digital Micrograph* software from Gatan.

The lateral sizes of the grains were examined using high-resolution scanning electron microscopy (SEM) on a Zeiss LEO-1530 instrument equipped with a field-emission cathode.

## 3. Results and discussion   

### 3.1. Detection and identification of lateral domains in polar GaN layers   

**Figure 1:**
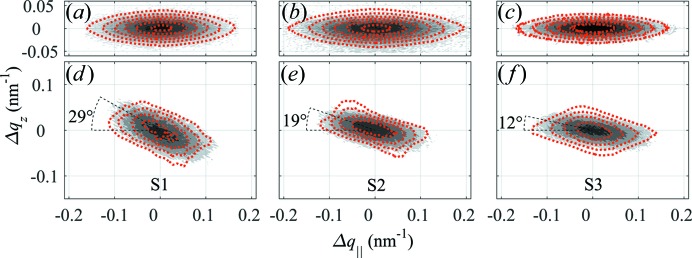
Comparison of experimental (grey filled contours) and simulated (red dotted contours) RSMs of reflections 004 [panels (*a*), (*b*) and (*c*)] and 114 [panels (*d*), (*e*) and (*f*)] for samples S1 [panels (*a*) and (*d*)], S2 [panels (*b*) and (*e*)] and S3 [panels (*c*) and (*f*)]. The intensities are plotted on a decimal logarithmic scale; the difference between adjacent contour lines is 10^0.5^. The angles displayed in panels (*d*)–(*f*) denote the inclinations of the measured 114 RLPs with respect to the *q*_||_ axis.

The 004 RSMs were used to determine the density of screw TDs as described by Barchuk *et al.* (2014 ▸). Within this approach, the measured RSMs are fitted by RSMs simulated using the kinematical theory of X-ray diffraction for different densities of screw TDs. The positions of the screw TDs are obtained using a Monte Carlo (MC) algorithm (Barchuk *et al.*, 2010 ▸). The results of the fits are presented in Figs. 1 ▸(*a*)–1 ▸(*c*). The corresponding densities of screw TDs (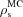) are listed in Table 1 ▸. The 114 RSMs were simulated with these screw TD densities and by changing the density of edge TDs (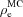) only. A comparison of measured and simulated 114 RSMs is displayed in Figs. 1 ▸(*d*)–1 ▸(*f*), and the densities of edge TDs obtained from the Monte Carlo simulation are summarized in Table 1 ▸.

**Figure 2:**
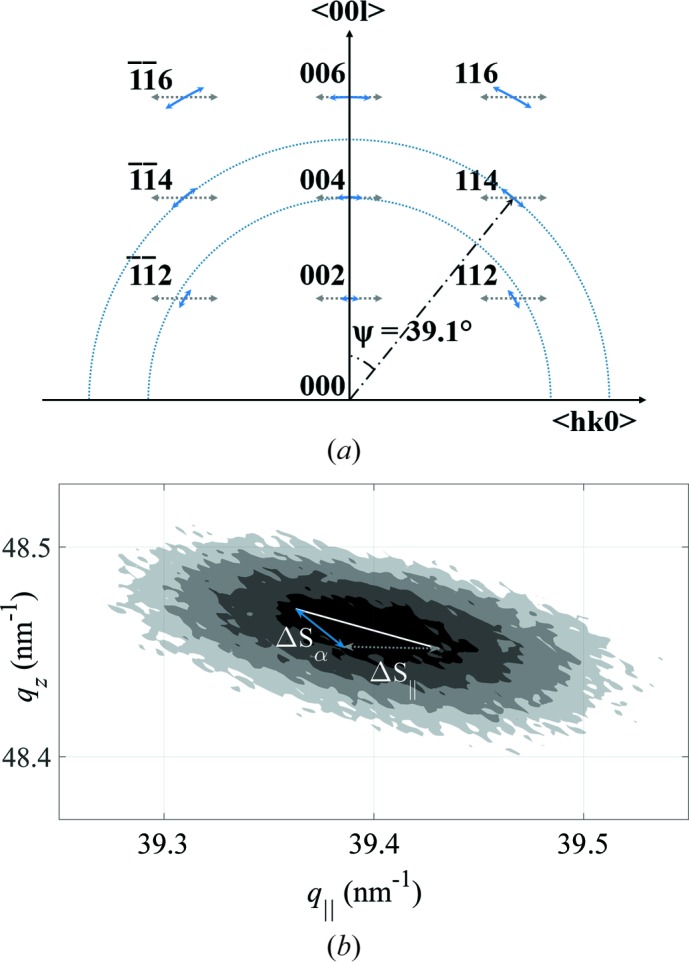
(*a*) Broadening of the RLPs due mainly to screw TDs (Δ*S*_α_, blue solid arrows) and the limited lateral size of the mosaic blocks (Δ*S*_||_, grey dotted arrows), depicted in reciprocal space. The broadening Δ*S*_α_ follows the Ewald sphere and scales with the length of the reciprocal-lattice vector, while Δ*S*_||_ is constant and always parallel to the *q*_||_ direction. Two Ewald hemispheres (blue dotted lines) are depicted for the measured diffractions 004 and 114. The angle ψ refers to the inclination of the reciprocal-lattice vector (114) from the surface normal (001). (*b*) The contributions Δ*S*_α_ and Δ*S*_||_ to the total broadening (white solid line) of the 114 RSM for sample S2. The intensities are plotted on a decimal logarithmic scale; the difference between adjacent contour lines is 10^0.5^.

For GaN layers grown by HTVPE, the 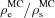 ratio typically approaches 5 (Barchuk *et al.*, 2016 ▸). This value was only observed for sample S1. For the other samples, the 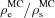 ratio is much smaller (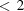) owing to the relatively high density of screw TDs, while the density of edge TDs does not change dramatically (Table 1 ▸). This result contradicts previous observations for GaN layers grown on Al_2_O_3_ substrates (Kaganer *et al.*, 2005 ▸, 2009 ▸; Barchuk *et al.*, 2014 ▸, 2016 ▸). Furthermore, analysis of the 114 RSMs for individual samples revealed that the directions of the dominant line broadening are inclined differently with respect to the direction of the diffraction vector (see the ξ values in Table 1 ▸). According to the usual mosaicity models (Holý *et al.*, 1993 ▸; Chierchia *et al.*, 2001 ▸; Moram & Vickers, 2009 ▸), TDs mainly cause a tilt of individual mosaic blocks, which produces azimuthal broadening of the RSMs (Fig. 2 ▸ *a*). Thus, the RSMs should be broadened in a direction perpendicular to the diffraction vector, which is inclined at 39.1° from the [001] direction for the GaN 114 reflection, whereas the values obtained from the measured RSMs range between 12° and 29°.

**Figure 3:**
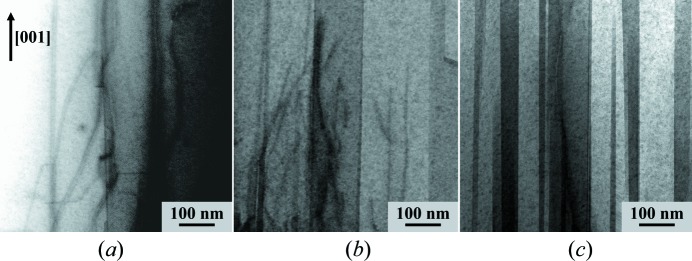
BF scanning TEM images of samples (*a*) S1, (*b*) S2 and (*c*) S3, containing TDs (dark bent lines, mainly in S1 and S2) and inverse domains (vertical stripes with different contrasts, mainly in S3).

In contrast with this tilt of individual mosaic blocks, which causes azimuthal broadening of RLPs, the limited lateral size of the domains gives rise to line broadening along the *q*_||_ direction (Fig. 2 ▸ *a*). When these two phenomena are superimposed, the RPLs are broadened in a direction which is inclined from the azimuthal direction towards the horizontal direction. Consequently, the inclination ξ depends on the ratio between these two contributions to the RLP broadening (Chierchia *et al.*, 2001 ▸). As the directions of the dominant broadening related to the respective effect are known, these two contributions can be separated graphically (see Fig. 2 ▸ *b*). For sample S2, Δ*S*_||_ is 4.5 × 10^−2^ nm^−1^ and the corresponding lateral crystallite size estimated using *D* = 2π/Δ*S*_||_ is 140 nm. The crystallite sizes determined in other samples are given in Table 1 ▸. Comparison with the results of SEM (Table 1 ▸) shows that objects having this lateral size cannot be columnar grains. On the contrary, the lateral sizes of the coherently diffracting domains (crystallites) agree well with the lateral sizes of the objects visualized by bright-field scanning TEM (Fig. 3 ▸).

In the bright-field scanning TEM micrographs, these objects appear as stripes with different contrast that propagate along the [001] direction, while the concurrently present TDs appear as dark bent lines propagating towards the sample surface. In sample S1, the density of the domain boundaries was low. Thus, it was not possible to determine the size of the lateral domains reliably from the TEM micrographs. In samples S2 and S3, the lateral sizes of the domains are 50–200 and 10–100 nm, respectively. From Table 1 ▸, the sizes of crystallites determined from the 114 RSMs are 140 and 75 nm for samples S2 and S3, respectively, which are in good agreement with the TEM data.

**Figure 4:**
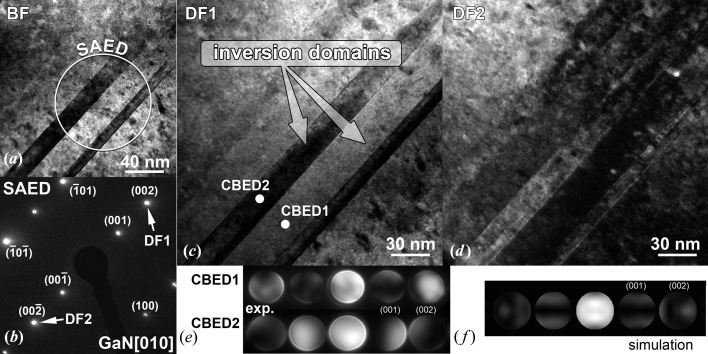
(*a*) BF TEM image of inversion domains in sample 3 and (*b*) the corresponding SAED pattern. (*c*), (*d*) DF TEM images in two beam conditions, (*c*) **g** = (002) and (*d*) **g** = (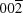), with the corresponding (*e*) experimental and (*f*) simulated CBED patterns.

In order to explain the nature of these domains, conventional BF TEM images of sample S3 were collected that comprise regions which include several vertical columns (Fig. 4 ▸ *a*). In contrast with the TDs, the walls of the domains did not bend, but stayed perfectly perpendicular to the sample surface, *i.e.* parallel to the [001] direction, across the whole layer. The SAED pattern recorded from the region inside the white circle in Fig. 4 ▸(*a*) is shown in Fig. 4 ▸(*b*). The diffraction spots do not reveal any significant broadening. Therefore, the presence of prismatic stacking faults, which are commonly observed in GaN (Northrup, 1998 ▸), can be excluded. Two DF TEM images recorded with opposite diffraction vectors, **g** = (002) and **g** = (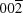), show opposite contrast of the stripes (see Figs. 4 ▸ *c* and 4 ▸ *d*, respectively), which provides evidence that these columns belong to domains with inverse polarities (Potin *et al.*, 1997 ▸; Potin, Ruterana & Nouet, 1999 ▸; Ruterana, 2005 ▸).

The presence of domains with inverse polarities was confirmed by CBED. The patterns shown in Fig. 4 ▸(*e*) were recorded from two domains with inverse polarities, and the investigated areas are marked by two white dots in Fig. 4 ▸(*c*). The contrasts of the diffraction spots in CBED1 and CBED2 are reversed (except for the central maximum 000), as expected for CBED patterns from inverse domains (Ruterana, 2005 ▸; Romano *et al.*, 1996 ▸). Since the CBED technique is very sensitive to the path of scattered electrons within the material and to local deformation fields, the dark and light contrasts in CBED patterns depend on the thickness of the specimen and local microstrains. Comparing pattern CBED1 from Fig. 4 ▸(*e*) with the CBED pattern (Fig. 4 ▸ *f*) simulated for a 200 nm thick specimen using the *JEMS* software from Stadelmann (1987 ▸), we can state that domain CBED1 has Ga-face polarity, whereas domain CBED2 is possibly N-face polar. The differences between the measured and simulated intensities of the CBED patterns are mainly due to a bowing of the TEM lamella induced during its preparation.

### 3.2. Effect of IDBs on the calculated densities of TDs   

IDBs disturb the coherence of polar GaN layers for X-ray scattering in the lateral direction, which leads according to Fig. 2 ▸ to the broadening of the RLPs along the *q*_||_ (or *hk*0) direction. This RLP broadening is superimposed on other contributions, which stem mainly from TDs. Note that screw TDs also broaden the RLPs in the *q*_||_ (or *hk*0) direction. When the GaN layers contain concurrently both TDs and IDBs (Fig. 3 ▸), the 00*l* RSMs are broadened in the *q*_||_ direction by both kinds of microstructure defect. The line broadening (in reciprocal-space units) stemming from TDs scales with the magnitude of the diffraction vector, whereas the additional broadening of RLPs caused by IDBs is constant (Fig. 2 ▸ *a*). Nevertheless, the line broadening from TDs cannot be reliably distinguished from the line broadening from IDBs if only a low number of RLPs are examined. Consequently, the density of screw TDs is overestimated if the effect of the IDBs is not taken into account.

In order to be able to distinguish the contributions of TDs and IDBs present concurrently in GaN samples (Fig. 3 ▸) to the measured line broadening, several RLPs must be analysed, as was suggested by Metzger *et al.* (1998 ▸) and Chierchia *et al.* (2003 ▸), within the mosaicity model (MM) which takes into account the line broadening from dislocations and from the finite lateral size of the crystallites. The densities of screw TDs (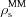), which were determined from the FWHMs of sample scans 002, 004 and 006 using the mosaicity model (Metzger *et al.*, 1998 ▸; Chierchia *et al.*, 2003 ▸), are lower than the TD densities obtained from the fitting of a single RSM (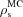) (see Table 1 ▸). This finding agrees well with the results of Lazarev, Bauer *et al.* (2013 ▸) and Lazarev, Barchuk *et al.* (2013 ▸), who also compared the TD densities obtained from a Monte Carlo simulation with those calculated from the mosaicity model and found 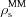 to be lower than 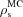.

In principle, analysis of the broadening of the 00*l* sample scans using the mosaicity model should be capable of revealing the lateral size of the crystallites (Metzger *et al.*, 1998 ▸; Chierchia *et al.*, 2003 ▸). However, this method obviously fails if the studied layers contain IDBs. For example, the lateral coherence length determined using the mosaicity model in sample S3 was *L*_||_ ≃ 700 ± 200 nm, which is much larger than the IDB width of 10–100 nm obtained from TEM or the lateral crystallite size of 75 ± 3 nm determined from asymmetric RSMs (see Table 1 ▸).

In the RSMs measured in asymmetric reflections, the superposition of line broadening from TDs and IDBs leads to an inclination of the intensity ridge, which substantially reduces the agreement between intensities in the simulated and measured RSMs, as can be seen in particular on the 114 RSMs in Fig. 1 ▸(*f*). Consequently, the inclination of the intensity ridge negatively affects the reliability of the edge TD densities obtained from a Monte Carlo simulation if the inclination of the asymmetric RSMs due to the presence of IDBs is not taken into account. On the other hand, the inclination of asymmetric RSMs from the expected direction, *i.e.* from a direction perpendicular to the diffraction vector in the case of TDs, can be used to estimate directly the contribution of IDBs to line broadening and the sizes of the GaN domains with inverse polarities.

## 4. Conclusions   

The influence of inversion domain boundaries on the shape of reciprocal space maps and on the densities of edge and screw threading dislocations determined using the mosaicity model and the Monte Carlo approach was illustrated on the example of polar GaN layers grown by high-temperature vapour phase epitaxy on [001]-oriented sapphire substrates. It was shown that IDBs interrupt the lateral coherence of GaN layers and consequently broaden the reciprocal-lattice points in the *q*_||_ direction. This effect can be directly recognized by the inclination of asymmetric RSMs from the direction perpendicular to the respective reciprocal-lattice vector. In contrast, the contributions of IDBs and TDs to the broadening of symmetric RLPs, which are 00*l* for *c*-oriented GaN, cannot be distinguished for single diffraction lines. Even if several 00*l* RSMs are taken into consideration, the contributions from IDBs and TDs to the RSM broadening cannot be separated with the same reliability as for asymmetric RSMs.

Therefore, the densities of screw TDs, which are calculated from the broadening of 00*l* reflections, are overestimated if the samples contain IDBs. The presence of IDBs as relevant microstructure defects was proven by bright-field and dark-field transmission electron microscopy, and by convergent-beam electron diffraction. Transmission electron microscopy revealed the width of domains with different polarities. Finally, it was shown that the width of incoherent GaN domains having different polarities can be reasonably estimated from the inclination of the intensity ridge in asymmetric RSMs.

## Figures and Tables

**Table 1:** The average lateral grain and crystallite sizes, the densities of screw TDs (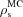) and edge TDs (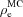) and their ratio (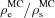) from the Monte Carlo simulation, the inclination of the RLPs with respect to the *q*_||_ axis (ξ), and the density of screw TDs (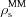) from the mosaicity model The grain size was determined using SEM and the other parameters using XRD performed on reflections 004 and 114. The theoretical value of ξ calculated for RSM 114 and TDs only is 39.1°.

Sample	Grain size (µm)	Crystallite size (nm)	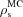 (× 10^9^ cm^−2^)	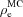 (× 10^9^ cm^−2^)	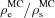	ξ (114) (°)	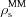 (× 10^9^ cm^−2^)
S1	4.7 ± 1.5	190 ± 6	0.8 ± 0.2	4.1 ± 1.0	5.1 ± 2.0	29	0.5
S2	1.5 ± 0.4	140 ± 4	2.1 ± 0.4	3.3 ± 0.8	1.6 ± 0.5	19	1.1
S3	1.7 ± 0.6	75 ± 3	1.6 ± 0.3	3.1 ± 0.8	1.9 ± 0.6	12	0.6
